# Adding Biotic Interactions into Paleodistribution Models: A Host-Cleptoparasite Complex of Neotropical Orchid Bees

**DOI:** 10.1371/journal.pone.0129890

**Published:** 2015-06-12

**Authors:** Daniel Paiva Silva, Sara Varela, André Nemésio, Paulo De Marco

**Affiliations:** 1 Instituto de Ciências Biológicas, Universidade Federal do Pará, Rua Augusto Corrêia, Guamá, Belém–PA, Brazil; 2 Museum für Naturkunde. Leibniz Institute for Evolution and Biodiversity Science. Invalidenstr. 43, Berlin, Germany; 3 Instituto de Biologia, Universidade Federal de Uberlândia–UFU, Rua Ceará, Campus Umuarama, Uberlândia–MG, Brazil; 4 Theory, Metapopulation, and Landscape Lab, Departamento de Ecologia, Instituto de Ciências Biológicas, Universidade Federal de Goiás, Campus II, Goiânia, Brazil; Field Museum of Natural History, UNITED STATES

## Abstract

Orchid bees compose an exclusive Neotropical pollinators group, with bright body coloration. Several of those species build their own nests, while others are reported as nest cleptoparasites. Here, the objective was to evaluate whether the inclusion of a strong biotic interaction, such as the presence of a host species, improved the ability of species distribution models (SDMs) to predict the geographic range of the cleptoparasite species. The target species were *Aglae caerulea* and its host species *Eulaema nigrita*. Additionally, since *A*. *caerulea* is more frequently found in the Amazon rather than the Cerrado areas, a secondary objective was to evaluate whether this species is increasing or decreasing its distribution given South American past and current climatic conditions. SDMs methods (Maxent and Bioclim), in addition with current and past South American climatic conditions, as well as the occurrences for *A*. *caerulea* and *E*. *nigrita* were used to generate the distribution models. The distribution of *A*. *caerulea* was generated with and without the inclusion of the distribution of *E*. *nigrita* as a predictor variable. The results indicate *A*. *caerulea* was barely affected by past climatic conditions and the populations from the Cerrado savanna could be at least 21,000 years old (the last glacial maximum), as well as the Amazonian ones. On the other hand, in this study, the inclusion of the host-cleptoparasite interaction complex did not statistically improve the quality of the produced models, which means that the geographic range of this cleptoparasite species is mainly constrained by climate and not by the presence of the host species. Nonetheless, this could also be caused by unknown complexes of other Euglossini hosts with *A*. *caerulea*, which still are still needed to be described by science.

## Introduction

Associations between host and parasitic species among Neotropical orchid bees (Hymenoptera: Apidae: Euglossini) have been mostly established based on casual and fortuitous reports (revised by [1,2]). Two genera among orchid bees comprise exclusively cleptoparasitic species: the monotypic *Aglae* Lepeletier & Serville, and *Exaerete* Hoffmannsegg, comprising eight species [3]. Besides field observations of cleptoparasitism, such behavior can also be inferred from particular morphological features of females of the parasitic species, such as the lack of corbiculae, a synapomorphic character shared by females of all non-parasitic Apini, Bombini, Euglossini and Meliponini, used in harvesting pollen and returning it to the nest or hive [4]. Species belonging to these two genera, *Aglae* and *Exaerete*, are known to be cleptoparasites on the nests of *Eufriesea* Cockerell and *Eulaema* Lepeletier species (revised by [2]).

Usually considered as an Amazonian orchid bee, *Aglae caerulea* Lepeletier & Servile (Apidae: Euglossini) has been recently reported within several savannic areas [5–7]. Although not widely distributed, individuals of this species are not particularly rare where the species occur (e.g. [8]). At least 15 species of *Eulaema* and 20 species of *Eufriesea* occur sympatrically with *A*. *caerulea*, but it has been only reported as an obligatory cleptoparasite on the nests of *Eulaema nigrita* Lepeletier, based on a single anedoctal record by J. G. Myers, in 1935 [9]. *Eulaema nigrita* is a large-bodied orchid bee which pollinates several different plant species [10–12] presenting a wide distribution in the Neotropics, ranging from Central America to southern Brazil and Argentina, being usually associated with fragmented and disturbed areas [13–16]. The aforementioned new occurrences for *A*. *caerulea* in savannic areas in Central Brazil raised doubts whether its populations are expanding their ranges towards central South America or are retreating their distribution backwards to the Amazon after several expansion and contraction events of this forest formation in previous moments of South America climatic history [17–23].

Here, species distribution models (SDMs hereon) were used to predict the potential distribution of a cleptoparasite orchid bee considering the distribution of its host as a predictor variable on both current and past scenarios of climatic conditions. Climatic conditions are one of the main factors determining species distributions, and thus, their inter-specific relationships [24–27]. Although South America was not covered by ice in any of the known glacial events that occurred in the planet, the climatic conditions of the Last Glacial Maximum (LGM hereon) were different from the current climate, and with an expected impact on the ranges of species [28,29]. Despite previous warmer moments, where the Amazonian was interconnected to the Atlantic forest, the palynological and vegetation evidences [18,30–33] show such connection was not reestablished with the temperature rise after the LGM, around five thousand years ago. The same period when the dry South American diagonal last developed with the interconnection of the current biomes of Caatinga, Cerrado, and Chaco [34,35].

Paleodistribution modelling has been successfully employed to analyze Quaternary megafaunal extinction events [36,37] and these methods are usually considered interesting tools to predict past species ranges [38,39]. These models correlate known occurrences of the focus species with environmental variables to create a multidimensional environmental space. Then, the species potential distribution is projected onto areas in the geographic space with similar environmental conditions to those found in the known occurrences [40].

Despite the advantages involved with the use of SDMs, they usually do not include biotic interactions to determine species ranges [41,42], an important determinant of species occurrences elsewhere [43]. Biotic interactions could vary from extremely positive interactions (e.g. mutualism) to extremely negative interactions (e.g. predation). Positive interactions could allow species to inhabit areas outside their own physiologic limits, while negative interactions (e.g. competition, parasitism, predation) could prevent species from inhabiting environmental favorable areas [44,45]. Many studies have already been developed on the attempt to integrate biotic interactions and species potential distributions, with results on the predictions of the potential distributions of the modeled species varying from significant improvement [46–49], mild or no improvement [50], and even decreases in the predictions of the modeled species [51].

Here, two different SDMs, Bioclim and Maxent, were used to predict the potential distribution of *A*. *caerulea* and *E*. *nigrita*. Two different models for *A*. *caerulea* were constructed, one based on the climatic conditions, and a second one in which the strong biotic interaction between the species was included by using the presence of *E*. *nigrita* as a predictor in the model of *A*. *caerulea*. Specifically, the study’s aims were: 1) to understand the role of Quaternary climatic changes on the distribution of both species; 2) to determine whether the recently reported occurrences of *A*. *caerulea* from savannic areas in Central Brazil are recent expansions from the Amazonian populations, or on the contrary, whether they are ancient populations of the species; 3) to test whether the map predictions for *A*. *caerulea* change after including a strong biotic interaction like host-parasite dependence in the model. As far as we are concerned, this is the first attempt to integrate host-parasite interactions across time using hindcast predictions with paleodistribution modeling.

## Methods

### Species occurrences

The casual sampling of new occurrences for *A*. *caerulea* used here, that generated the question whether this species was increasing or decreasing its current distribution when compared to the its past one, were the same used in Silva *et al*. [7]. However, additional occurrences obtained from Abrahamczyk *et al*. [52] and Freitas and Nemésio [6] were included here. The occurrences of *E*. *nigrita* used here were obtained from CRIA’s Species Link (www.splink.org.br) and GBIF (www.gbif.org), with the same methods employed by Silva *et al*. [7] to georeference occurrences. For those occurrences with reliable geographic information, but lacking specific latitude/longitude information, Google Earth [53] was employed to acquire municipality geographic information, later used in the modeling procedures. Additional occurrences were obtained in the published literature, as shown in the Supplementary Material (S1 Text). The samplings of new occurrecens for *A*. *caeruea* in Silva *et al*. [7] occurred in particular areas with the consent of the property’s owner. The occurrence data for both host and cleptoparasite bees are available in the supplementary material (S1 File and S2 File).

### Climatic variables

Worldclim’s current variables and CCSM and MIROC General Circulation Models Worldclim variables (www.worldclim.org) at 2.5 minutes resolution were used to produce the different models. Five variables out of the 19 variables available from WorldClim [54] were selected to produce the distribution models: BIO 4 (Temperature Seasonality (standard deviation *100)), BIO8 (Mean Temperature of Wettest Quarter), BIO9 (Mean Temperature of Driest Quarter), BIO16 (Precipitation of Wettest Quarter) and BIO17 (Precipitation of Driest Quarter). Temperature seasonality was used because this variable has been related to the distribution of euglossine species [52]. Temperature and precipitation of the driest and the wettest quarter were also considered. In tropical and subtropical South America, the dry and the wet season clearly influence the biotic cycles of these orchid bee species [52,55].

### Ecological niche models

Two different algorithms, Maxent [56,57] and Bioclim [58] were selected to produce the potential distributions. Those two models are different approaches for predicting the distribution of species when the data sample has no real absences (necessary if logistic regressions or classification techniques are required; [59]). Maxent model fits an algorithm to separate the occurrence records from the background conditions [60], while Bioclim map the percentile distribution of the occurrences in the studied extent [61].

Maxent and Bioclim model predictions could be sensitive to bias and errors in the data [62]. For that reason, species occurrences have been filtered to select a balanced calibration data set [63,64]. The selected calibration data sets should be balanced in the environmental space, but not necessarily in the geographic space (geographically close occurrences could have different and thus, important information for the model, while geographically separated occurrences could have similar environmental information). The envSamp R-function (https://github.com/SaraVarela/envSample) was used to filter the data. Three environmental filters were set: BIO4 (Temperature seasonality: standard deviation *100) = 200, BIO16 (Precipitation of Wettest Quarter) = 200 mm, BIO17 (Precipitation of Driest Quarter) = 200 mm. By doing so, 25 out of the 44 presumably biased raw occurrences for *A*. *caerulea* were used (S1 Fig). The 19 discarded points were used to test the model predictions. In addition, the same filter was also applied to the 290 raw occurrences from *E*. *nigrita*, and 66 balanced occurrences were selected to calibrate the models (S2 Fig). The 224 discarded occurrences were used to test the model predictions. The *Area Under the receiver-operator Curve* (AUC hereon) was calculated as a measure of the discriminative power of the Maxent results. The AUC values for *A*. *caerulea* could be compared between models because the same training and testing occurrence records were used, plus the same geographic extent to train the Maxent models [65].

For constructing the models, the functions *Bioclim* and *Maxent* from the *dismo* R-package [66] were used. The model using the filtered occurrences (see above) was calibrated to overcome the potential biases of the raw occurrence dataset. For Maxent, the model was calibrated using the filtered occurrences, plus 10,000 background points from the study extent (South America). Model overfitting was avoided by setting the argument betamultiplier = 2, not allowing the algorithm to construct a model using *ad-hoc* and complex equations [67]. To evaluate the models, the occurrences discarded from the training dataset were used, plus the same number of absences selected randomly from the study extent. Thus, since the number of occurrences and pseudoabsences were the same, the default value of prevalence 0.5 was used to evaluate the model predictions [65]. Other parameters not mentioned here were set as default.

Finally, the threshold that maximizes both sensitivity and specificity was used [62,68], for constructing the binary predictions for Bioclim and Maxent. The LGM maps are the result of averaging the predictions from the two general circulation models (CCSM and MIROC) and, subsequently, using the threshold to obtain the binary prediction. Omission errors (percentage of errors in the prediction of presences) were calculated. Neither commission errors nor True Skilled Statistics were calculated because there were no real georeferenced absences for either of the species considered. Consequently, the Akaike Criterion (AIC) and the relative likelihood (exp((AIC_min_O-OAIC_j_/2)) were used in order to compare the models for *A*. *caerulea*, after including and excluding the models of *E*. *nigrita*, its host, as a predictor variable. AIC is a measure of the quality of a model given a set of data [69] and these methods were done using ENMeval [70]. All R-scripts are available in https://github.com/SaraVarela/host_parasite_ENM.

## Results

**Fig 1:**
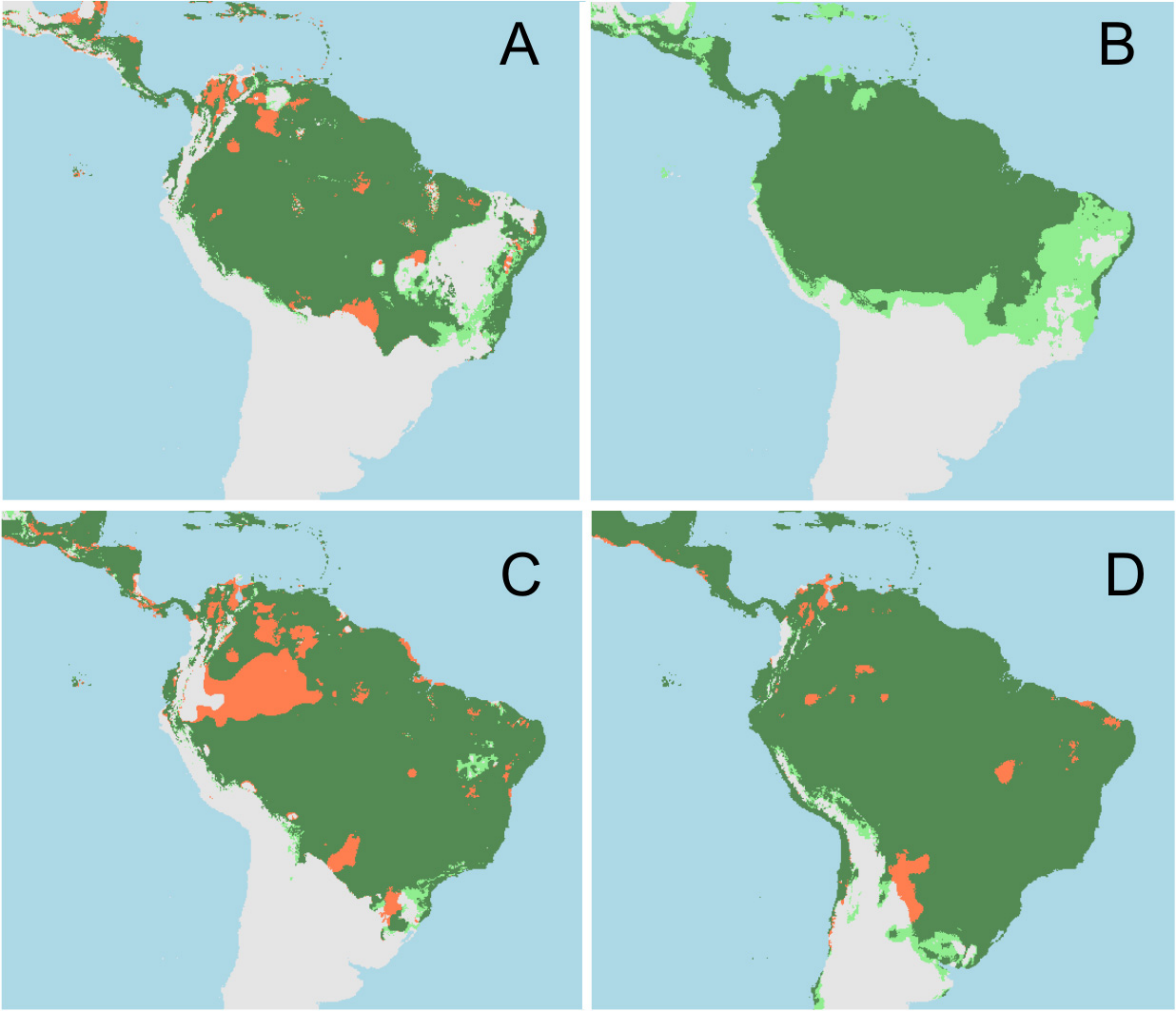
Potential range shift of the species as a consequence of the climatic changes of the last 21,000 years. Stable areas for both species are in dark green, areas predicted suitable during the Last Glacial Maximum are in red, and new expansions (younger than 21,000 years) in pale green. A) *Aglae caerulea* distribution according to Bioclim. B) *Aglae caerulea* distribution according to Maxent; C) *Eulaema nigrita* distribution according to Bioclim, D) *Eulaema nigrita* distribution according to Maxent. Climatic changes of the last 21,000 years did not strongly affect any of these species.

Precipitation of the wettest quarter (BIO16), Temperature seasonality (BIO4), and Precipitation of the driest quarter (BIO17) determined the distribution of *A*. *caerulea*, based in the Maxent results (Table 1). Both training and testing AUC values for the models considering the distribution of *A*. *caerulea* were high, reaching 0.79. Omission rate (percentage of errors in the occurrences) was equal to 0, what means that the occurrences of *A*. *caerulea* are being perfectly predicted. Thus, the model was able to predict both training and testing datasets. Maxent and Bioclim models predicted that the distribution of *A*. *caerulea* was not strongly affected by the LGM (Fig 1A and 1B). Following the results, current populations of *A*. *caerulea* from the Brazilian Cerrado could be as old as Amazonian populations.

**Table 1:** Maxent model results for *A*. *caerulea*, using only climatic variables to calibrate the model.

Model training	
Training occurrences	25
Training background data	10025
Beta multiplier	2
Iterations	260
Training AUC	0.790
Individual contribution of the variables	
bio16	45.819
bio17	12.626
bio4	41.554
bio8	0
bio9	0
Permutation importance of the variables	
bio16	60.076
bio17	11.607
bio4	28.316
bio8	0
bio9	0
Model testing	
Testing occurrences	19
Testing absence data	19
Prevalence	0.500
Testing AUC	0.790

Temperature seasonality, Mean Temperature of Wettest Quarter, Precipitation of the driest quarter, and Mean Temperature of driest Quarter were the four variables that determined the distribution of *E*. *nigrita* according to Maxent results (Table 2). AUC reached 0.76 for the training data set and 0.75 for the testing data set. Thus, the model had a good discriminative power for the training and the testing datasets. In this case, omission rate was 0.02, what means that the presences of *E*. *nigrita* are also being perfectly predicted by the models. Bioclim and Maxent models predicted that *E*. *nigrita* have had a large potential suitable area in South America during both, glacial and interglacial periods (Fig 1C and 1D). The overlap of *A*. *caerulea* with its host, *E*. *nigrita*, was very similar from the LGM to the present, showing only 0.02% of change between each climatic scenario.

**Table 2:** Maxent model results for *E*. *nigrita*, using only climatic variables to calibrate the model.

Model training	
Training occurrences	66
Training background data	10066
Beta multiplier	2
Iterations	500
Training AUC	0.760
Individual contribution of the variables	
bio16	0.914
bio17	14.927
bio4	46.306
bio8	23.601
bio9	14.250
Permutation importance of the variables	
bio16	4.622
bio17	14.320
bio4	55.012
bio8	26.044
bio9	0
Model testing	
Testing occurrences	224
Testing absence data	224
Prevalence	0.500
Testing AUC	0.750

**Fig 2:**
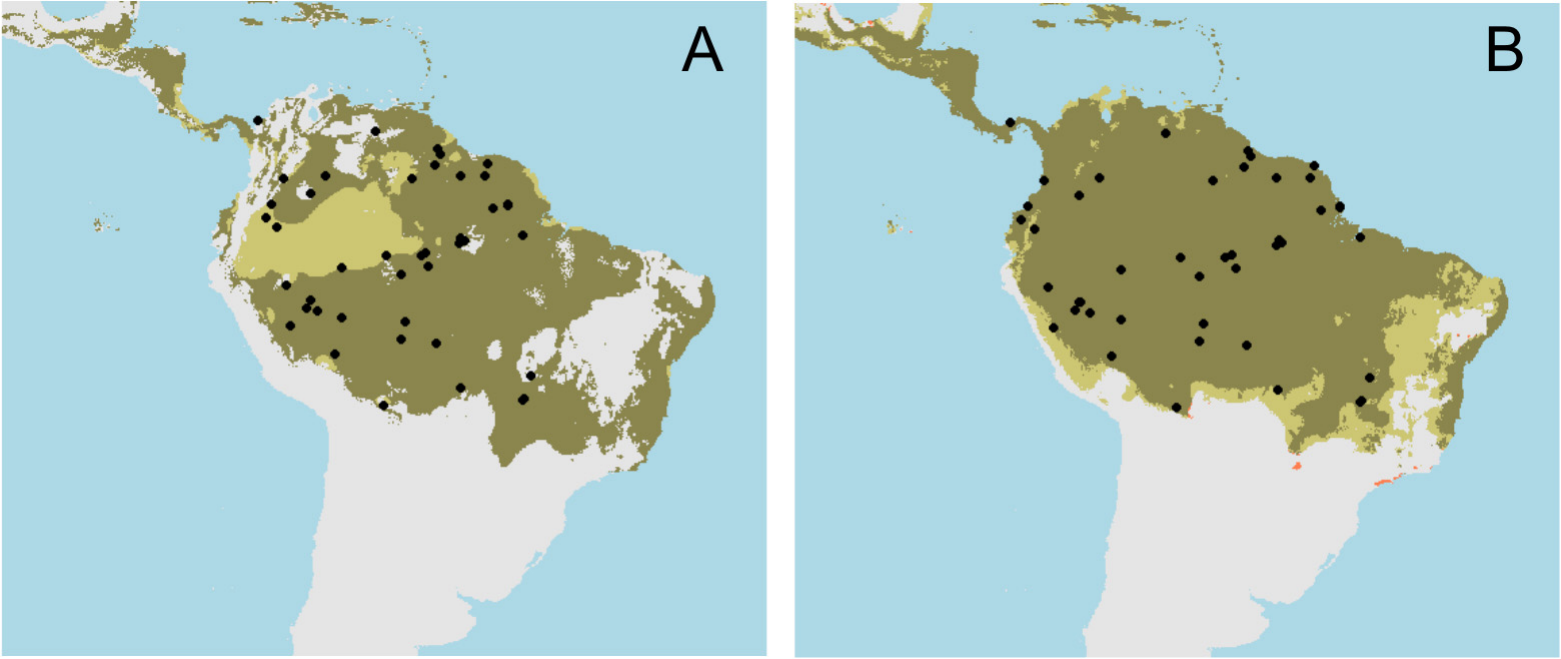
Predictions regarding the current distribution of *Aglae caerulea* according to A) Bioclim and B) Maxent. The areas in pale brown become unsuitable for the species after including the host species, *Eulaema nigrita*, as a predictor in the models. Points are the observed occurrences for *A*. *caerulea*. The main difference between the models is that Bioclim predictions with *E*. *nigrita* exclude the Western areas of the Amazonas and thus, when including this variable in the model for predicting *A*. *caerulea*, the resulting map shows this pattern.

The Maxent results showed that climatic variables are still the most important factors determining the distribution of *A*. *caerulea*, after the distribution of *E*. *nigrita* was considered as an environmental predictor (Table 3). Precipitation of the wettest quarter (BIO16), Temperature seasonality (BIO4), and Precipitation of the driest quarter (BIO17) determined the distribution of *A*. *caerulea* based in the Maxent results (Table 3). The presence of *E*. *nigrita* had a small contribution to the final model of *A*. *caerulea* (Table 3). Overall, this model had an omission rate of 0.03, what means that the presences are being perfectly predicted) and a lower discriminative power than the climatic models, with a training AUC equal to 0.70. However, it has a high predictive power in the test sample, with AUC equal to 0.85. These results indicate that the relative likelihood of both models is similar (the relative likelihood of the complete model with the host species is < 0.0001), and statistically, no distinction of the best model solution can be made. When the presence of *E*. *nigrita* was included as one predictor variable for modeling the distribution of *A*. *caerulea*, the map predictions decreased. Specifically, the Bioclim predictions excluded western portions of the Amazon as potential distribution areas for *A*. *caerulea* (Fig 2A). Conversely, in the predictions generated by Maxent, eastern areas for *A*. *caerulea* became unstable (Fig 2B). Nonetheless, as *E*. *nigrita* has a broad suitable range, the potential distribution of *A*. *caerulea* potential distribution was not strongly affected by its host species in any of the periods considered.

**Table 3:** Maxent model results for *A*. *caerulea*, using climatic variables plus the presence of *E*. *nigrita*, its host species, as a predictor variable.

Training the model	
Training occurrences	25
Training background data	10025
Beta multiplier	2
Iterations	420
Training AUC	0.700
Contribution of the variables	
bio16	45.800
bio17	18.235
bio4	35.031
bio8	0
bio9	0.250
Presence of *E*. *nigrita*	0.681
Permutation importance of the variables	
bio16	22.285
bio17	14.788
bio4	61.446
bio8	0
bio9	0.529
Presence of *E*. *nigrita*	0.950
Testing the model	
Testing occurrences	19
Testing absence data	19
Prevalence	0.500
Testing AUC	0.850

## Discussion

The predictions of the potential distribution of a host-parasite complex of South American orchid bees were assessed when the host species distribution was included as a predictor variable of the parasitic bee, in the current and past climatic conditions for the LGM event. Additionally, climatically stable areas for the potential distribution of *A*. *caerulea* in both periods were estimated, on an attempt to discover whether the newer distributional occurrences for this species in the Brazilian Cerrado savanna [5–7] are related to range increases or decreases of the species. Although the map predictions for the potential distribution of *A*. *caerulea* did not increase when the distribution of *E*. *nigrita* was considered as a predictor, suitable areas for *A*. *caerulea* in the Cerrado seemed to be as stable as the Amazonian ones. Additionally, some areas in the eastern Brazilian coast became climatically suitable for this species after the LGM. Finally, some areas became unsuitable for *A*. *caerulea* distribution in western Amazon and northeastern Brazil after the inclusion of the distribution of *E*. *nigrita* as a predictor variable determining the distribution of *A*. *caerulea*, depending on the algorithm considered.

Although ice sheets did not cover South America, past climatic changes had severe effects on South American forested areas (e.g. Amazon and Atlantic Forests) and their related biota [19–23]. Nonetheless, the distribution of *A*. *caerulea*, in both past and current climatic conditions, covers a similar area. Despite its usual xeric-like environment, the Brazilian Cerrado, the second largest biome in South America [33], has many evergreen gallery forests surrounding its rivers and streamlets. These habitats provide both environmental and biotic conditions for the maintenance of Amazonian fauna which disperse through the Cerrado biome, especially for Euglossini bees [71]. Consequently, this vegetation serve as an important dispersal route for *A*. *caerulea* [7] and may have exerted the same role in past contraction events of the Amazonian forest, also supporting the high climatic stability of *A*. *caerulea* distribution across time.

Nonetheless, host-parasite interactions may also explain the climatic stability observed for *A*. *caerulea* in this study. While *A*. *caerulea* is mainly distributed in Amazon with scattered occurrences in Cerrado, *E*. *nigrita* is a wide-ranged species, which benefits from and have higher abundances in opened areas or in anthropic areas [16,72,73]. The anecdotal report of this host-parasite complex occurred more than 80 years ago [9] and since then, *A*. *caerulea* has not been reported to parasitize nests of other orchid bees.

On the other hand, *E*. *nigrita* has been confirmed to be host to at least another widespread cleptoparasitic orchid bee, *Exaerete smaragdina* Guérin-Menéville, which is suggested to exert strong control on the populations of *E*. *nigtrita* [2]. This latter association may limit the availability of nests to be parasitized by *A*. *caerulea* in the region where the three species are sympatric, i.e., the Amazon and the Brazilian Cerrado. However, it should be stressed that *A*. *caerulea* might parasitize orchid bee species other than *E*. *nigrita*. Based on the relative frequencies of known host-parasite associations, Nemésio and Silveira (2) showed that some of these associations could not be exclusive, since the known parasitic species occurred in sites where the host had never been recorded or, alternatively, the relative frequency of the parasitic species was higher than that observed for the host species. The only explanation found by those authors to deal with such a discrepancy, considering the database is reliable, is that many parasitic species may have more than one host species. Almost 30 species of *Eulaema* and more than 60 species of *Eufriesea* are described from the Neotropical region [3]. Most of them have a similar size or are larger when compared to *A*. *caerulea* and many of them occur in sympatry with *A*. *caerulea*. Therefore, especially for those areas depicted as climatically unstable with the inclusion of *E*. *nigrita* as a predictor determining the distribution of *A*. *caerulea*, other orchid bees may serve as hosts for *A*. *caerulea*. Since host-parasite relationships in orchid bees are generally based on fortuitous observations [2], *Eulaema* nests other than *E*. *nigrita*, besides those of *Eufriesea* species, are plausible to be recorded as hosts for *A*. *caerulea* in future field studies aiming to establish such associations.

The use of biotic related variables in species distribution modeling are becoming increasingly frequent in macroecology, with variable results ranging from positive [46–49], neutral [50] to negative [51]. Even so, in this study, the inclusion of *E*. *nigrita* as a predictor of the distribution of *A*. *caerulea* did not increase the prediction of its potential distribution. Despite these results, the attempts to make distribution models more realistic, and consequently, encompass the biotic component of the Biotic-Abiotic-Migration (BAM) diagram [41,74] into the distribution models are interesting perspectives, especially if ongoing climate changes are considered. Many insect species rely on specific inter-specific biotic interactions (e.g. mutualism, parasitism) to survive and reproduce. Nonetheless, during historical and current scenarios of climate change, such interactions may uncouple or fail given spatial or phenological mismatches between the interacting species [75–79]. Therefore, further investigation on how interacting species will behave upon future climate changes are needed especially given concerning scenarios of biodiversity loss [80–82] for a better assessment on how will species interactions maintain themselves in the future. Similar approaches to those employed here, considering the potential range of interactions in different climatic scenarios, are already being done elsewhere and considering different biological groups and areas (e.g. [50,79,83,84]). These methods should be replicated either to obtain distribution models that are biologically more meaningful than those only using bioclimatic variables as well as to better understand the behavior of the SDMs when these biotic interactions are used as determinants of species distributions.

## Supporting Information

S1 Fig Distribution of the 44 occurrences for *Aglae caerulea* in A) the environmental space (Filter 1 = Temperature seasonality, Filter 2 = Mean temperature of wettest quarter), and in B) the geographic space. Occurrences selected for calibrating the model are depicted in red, while those selected for testing the model are in grey. Selection was based on envSample R-function (https://github.com/SaraVarela/envSample).
(DOC)

S2 Fig Distribution of the 290 occurrences for *Eulaema nigrita* in A) the environmental space (Filter 1 = Temperature seasonality, Filter 2 = Mean temperature of wettest quarter), and B) in the geographic space. The 66 occurrences selected for calibrating the model are depicted in red, while the 264 selected for testing the model are in grey. Selection was based on envSample R-function (https://github.com/SaraVarela/envSample).
(DOC)

S1 File Occurrence records for *Aglae caerulea*. (XLS)

S2 File Occurrence records for *Eulaema nigrita*. (XLS)

S1 Text Reference list of manuscripts holding occurrences of *E*. *nigrita*. (DOC)
