# Supplementary figures and images for: Adding Biotic Interactions into Paleodistribution Models: A Host-Cleptoparasite Complex of Neotropical Orchid Bees
Source: PLoS One. 2015 Jun 12;10(6):e0129890. doi: 10.1371/journal.pone.0129890 (PMC4466402; doi:10.1371/journal.pone.0129890)

| 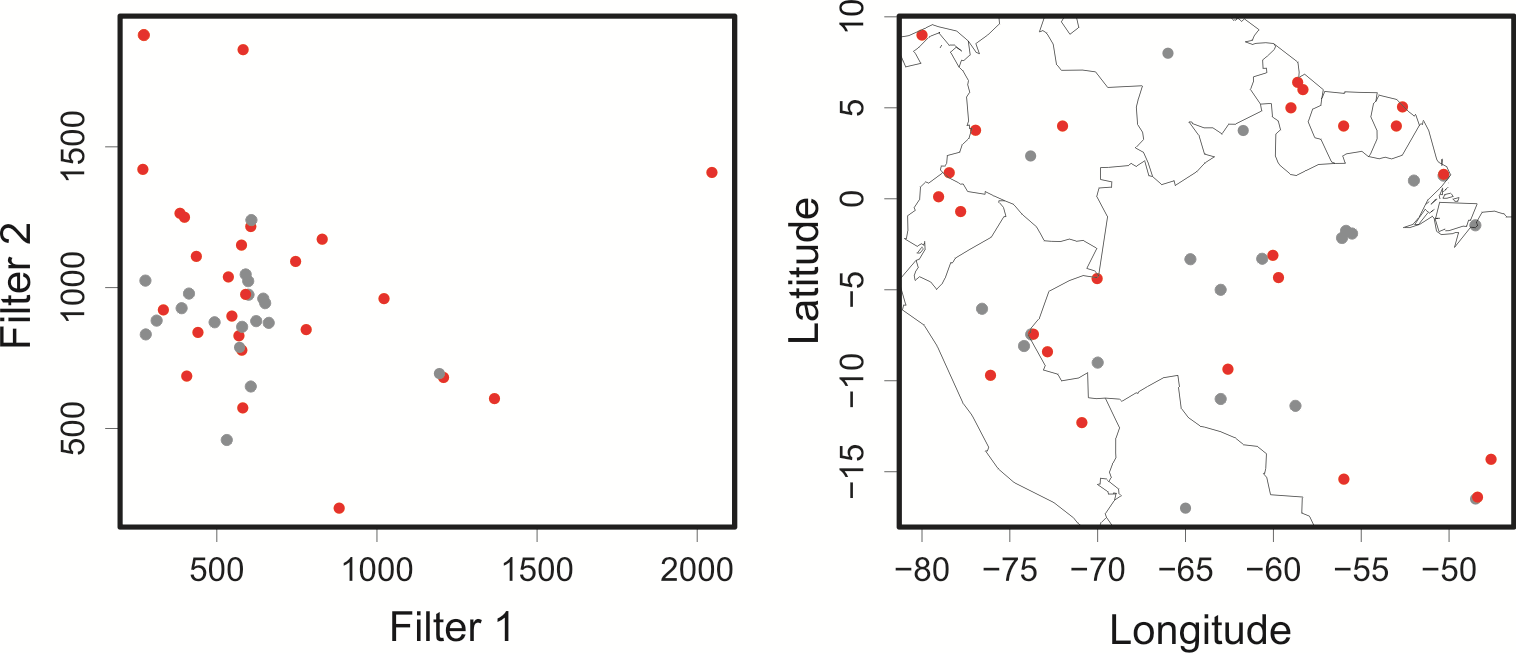 |
| --- |

Supplement: S1 Fig — Occurrences selected for calibrating the model are depicted in red, while those selected for testing the model are in grey. Selection was based on envSample R-function (https://github.com/SaraVarela/envSample). (DOC) [file pone.0129890.s001.doc]

| 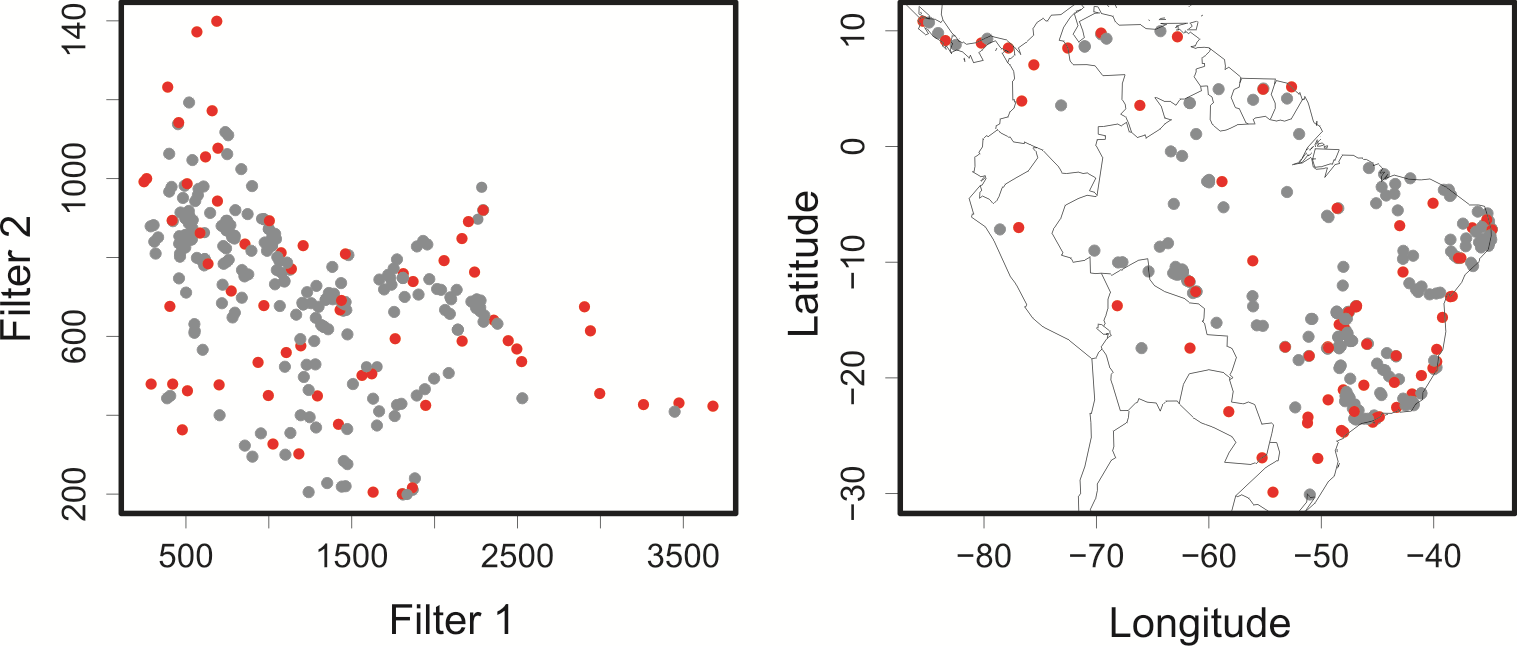 |
| --- |

Supplement: S2 Fig — The 66 occurrences selected for calibrating the model are depicted in red, while the 264 selected for testing the model are in grey. Selection was based on envSample R-function (https://github.com/SaraVarela/envSample). (DOC) [file pone.0129890.s002.doc]
