# Supplementary material for: Adding Biotic Interactions into Paleodistribution Models: A Host-Cleptoparasite Complex of Neotropical Orchid Bees
Source: PLoS One. 2015 Jun 12;10(6):e0129890. doi: 10.1371/journal.pone.0129890 (PMC4466402; doi:10.1371/journal.pone.0129890)
Supplement: S1 Text — (DOC) [file pone.0129890.s005.doc]

Aguiar, C., & Zanella, F. (2005). Estrutura da comunidade de abelhas (Hymenoptera: Apoidea: Apiformis) de uma área na margem do domínio da caatinga (Itatim, BA). Neotropical Entomology, 34(1), 15–24.

Silva, C. I., Augusto, S. C., Sofia, S. H., & Moscheta I. S. (2007). Diversidade de Abelhas em Tecoma stans (L.) Kunth (Bignoniaceae): A importância na Polinização e Produção de Frutos, Neotropical Entomology, 36(3), 331–341.

Aguiar, A. J. C., & Martins, C. F. (2003). The bee diversity of the tabuleiro vegetation in the Guaribas Biological Reserve (Mamanguape, Paraíba, Brazil). In: G.A. Melo & I. ALVES-DOS-SANTOS (eds.), Apoidea Neotropica: Homenagem aos 90 anos de Jesus Santiago Moure, 209-216.

Alvarenga, P. E. F., Freitas, R. F., & Augusto, S. C. (2007). Diversidade de Euglossini (Hymenoptera: Apidae) em áreas de Cerrado do Triângulo Mineiro, MG. Bioscience Journal, 23(1), 30–37.

Albuquerque et. al (2001). Levantamento da fauna de abelhas silvestres (hymenoptera, apoidea) na região da “baixada maranhense”: Vitória do Mearim, MA, Brasil. Acta Amazônica, 31(3), 419-430.

Antonini, Y., & Martins, R. (2003). The flowering-visiting bees at the ecological station of the Universidade Federal de Minas Gerais, Belo Horizonte, MG, Brazil. Neotropical Entomology, 32(4), 565–575.

Araújo, V. A, Antonini, Y., & Araújo, A. P. A. (2006). Diversity of bees and their floral resources at altitudinal areas in the Southern Espinhaço Range, Minas Gerais, Brazil. Neotropical Entomology, 35(1), 30–40.

Brito, C. M., & Rêgo, M. M. (2001). Community of male Euglossini bees (Hymenoptera: Apidae) in a secondary forest, Alcântara, MA, Brazil. Brazilian journal of biology, 61(4), 631–8.

Carvalho, C., Rêgo, M., & Mendes, F. (2006). Dinâmica de populações de Euglossina (Hymenoptera, Apidae) em mata ciliar, Urbano Santos, Maranhão, Brasil. Iheringia. Série Zoologia, 96(2), 249–256.

Alfredo, C., Carvalho, L., Silva, R. F., & Souza, B. D. A. (2007). Phenology of bees (Hymenoptera: Apoidea) in a transition area between the Cerrado and the Amazon region in Brazil. Sociobiology, 50(3), 1177-1900.

Freitas, R. F. D. E. (2009). Diversidade e sazonalidade de abelhas Euglossini latreille (Hymenoptera: Apidae) em fitofisionomias do bioma cerrado em Uberlândia , MG. Dissertação de Mestrado, Instituto de Biologia, Universidade Federal de Uberlândia.

Martins, C. F. (1994). Comunida de Abelhas (Hym., Apoidea) da Caatinga e do Cerrado com elementos de Campo rupestre do estado da Bahia, Brasil. Revista Nordestina de Biologia, 9, 225-257.

Mendes, F. N., Rêgo, M. M. C., & Carvalho, C. C. (2008). Abelhas Euglossina (Hymenoptera, Apidae) coletadas em uma monocultura de eucalipto circundada por Cerrado em Urbano Santos, Maranhão, Brasil. Iheringia*.* Série Zoologia, 98(3), 285–290.

Nemésio, A., &Faria-Junior, L. (2004). First assessment of the orchid-bee fauna (Hymenoptera: Apidae) at Parque Estadual do Rio Preto, a cerrado area in southeastern Brazil. Lundiana, 5(2), 113–117.

Manuel, J., Rebelo, A. M., & Cabral, J. (1997). Abelhas euglossinae de barreirinhas, zona do litoral da baixada oriental maranhense. Acta Amazônica, 27(2), 145–152.

Santiago, L. R., Mahlmann, T., Lopes, V., & Santana, F.. (2009). A fauna apícola do Parque Municipal da Cachoeirinha (Iporá, GO). Biota Neotropical, 9(3), 1–5.

Milet-Pinheiro, P.& Schlindwein, C. (2005). Do euglossine males (Apidae, Euglossini) leave tropical rainforest to collect fragrances in sugarcane monocultures? Revista Brasileira de Zoologia, 22(4), 853–858.

Andena, S., Bego, L., & Mechi, M. (2008). A comunidade de abelhas (Hymenoptera, Apoidea) de uma área de cerrado (Corumbataí, SP) e suas visitas às flores. Revista Brasileira de Zoociências, 7(1), 55–91

.

Carlos, P. (2003). Variação do tamanho corporal de machos de Eulaema nigrita Lepeletier (Hymenoptera, Apidae, Euglossini). Resposta materna à flutuação de recursos? Revista Brasileira de Zoologia, 2(1969), 207–212.

Silva, O. L. (2009). Análises filogeograficas de *Exarete Smaragdina* (Guérin-Méneville,1845) (Hymenoptera, Apidae, Euglossini) e sua hospedeira *Eulaema nigrita* (Lepeletier, 1841) (Hymenoptera, Apidae, Euglossini) e o status *de Exarete Lepeletieri* (Oliveira e Nemésio, 2003). Dissertação de Mestrado, Faculdade Federal de São Carlos, 57p.

Aguiar, W., & Gaglianone, M. (2012). Euglossine bee communities in small forest fragments of the Atlantic Forest, Rio de Janeiro state, southeastern Brazil (Hymenoptera, Apidae). Revista Brasileira de Entomologia, 56(2), 210-219.

Farias, R. (2007). Euglossina (Hymenoptera: Apidae) e preferência por fragrâncias artificiais em mata e dunas na Área de Proteção Ambiental da Barra do Rio Mamanguape, Rio. Neotropical Entomology, 36(6), 863–867.

Viana, B. F., Kleinert, A. M. P., & Neves, E. L. (2002). Comunidade de Euglossini (Hymenoptera, Apidae) das dunas litorâneas do Abaeté, Salvador, Bahia, Brasil. Revista Brasileira de Entomologia, 46(4), 539–545.

Pedro, S.R.M. (1992). Sobre as abelhas (Hymenoptera, Apoidea) em um ecossistema de cerrado (Cajuru, NE do Estado de São Paulo): composição, fenologia e visita às flores. Dissertação de Mestrado, Faculdade de Filosofia, Ciências e Letras de Ribeirão Preto, Universidade de São Paulo.

Mateus, S. (2011). Abundância relativa, fenologia e visita as flores pelos Apoidea do cerrado da estação ecológica de Jataí-Luiz Antônio-SP. Dissertação de Mestrado, Universidade de São Paulo, 51p.

Azevedo, A.A. (2002). Composição de faunas de abelhas (Hymenoptera, Apoidea) e da flora associada em áreas de cerrado de Minas Gerais, Brasil. Dissertação de Mestrado, Universidade Federal de Viçosa, 50p.

Boaventura, M.C. (1998). Sazonalidade e Estrutura de uma comunidade de abelhas silvestres (Hymenoptera, Apoidea) numa área de cerrado do Jardim Botânico de Brasília, Distrito Federal. Dissertação de Mestrado, Universidade de Brasília, 99p.

Silveira, F.A. (1989). Abelhas silvestres (Hymenoptera: Apoidea) e suas fontes de alimento no cerrado da estaçao florestal de experimentaçao de Paraopeba - Minas Gerais. Dissertação de Mestrado, Universidade Federal de Viçosa, 50p

.

Faria Jr., L. R. R. (2005). Euglossina (Hymenoptera, Apidae) em áreas de cerrado s.s. e mata ciliar em Brasilândia de Minas, MG, com uma discussão sobre a biogeografia do grupo no cerrado. Dissertação de Mestrado, Universidade Federal de Minas Gerais, Belo Horizonte, 90p.

Aguilar, J. B. V. (1990). Contribuição ao conhecimento dos Euglossini (Hymenoptera: Apidae) do estado da Bahia, Brasil. Dissertação de Mestrado, Instituto de Biociências, Universidade de São Paulo, 96p.

Pinheiro-Machado, C. A. (2002). Diversidade e conservação de Apoidea: relações entre riquezas de espécies e alteração ambiental no Brasil. Dissertação de Mestrado, Universidade de São Paulo, 142p.

## List of institutions holding *E. nigrita*’s occurrences

1. Coleção de Abelhas do Museu de Ciências e Tecnologia da PUCRS
2. Coleção Entomológica Paulo Nogueira-Neto – IB/USP
3. ICN - Instituto de Ciencias Naturales
4. Instituto Nacional de Biodiversidad (INBio), Costa Rica
5. Universidad del Valle (Colômbia)
6. University of Kansas Biodiversity Institute
7. USDA-ARS Bee Biology and Systematics Laboratory
8. LEBIC - Laboratório de Ecologia e Biogeografia de Insetos da Caatinga
9. SINBIOTA - Sistema de Informação do Programa Biota/Fapesp
10. MZUEL-ABELHAS, Museu de Zoologia da Universidade Estadual de Londrina
11. CEPANN - Coleção Entomológica Paulo Nogueira-Neto - IB/USP
12. CE-UFPE, Coleção Entomológica da UFPE
13. CEMeC - Coleção Entomológica Moure & Costa
14. UFES-ENTOMOLOGIA, Coleção Entomológica da UFES
15. DZUP-HYMENOPTERA, Coleção Entomológica Pe. Jesus Santiago Moure (Hymenoptera)
16. DSEC - Coleção Entomológica do Depto. de Sistemática e Ecologia
17. INPA-HYMENOPTERA, Coleção de Hymenoptera INPA
